# Supplementary material for: Influence of Chromophoric Electron-Donating Groups on Photoinduced Solid-to-Liquid Transitions of Azopolymers
Source: Polymers (Basel). 2020 Apr 13;12(4):901. doi: 10.3390/polym12040901 (PMC7240690; doi:10.3390/polym12040901)
Supplement: Supplementary file 1 [file polymers-12-00901-s001.pdf]

polymers

Support Information

Influence of Chromophoric Electron-Donating Groups on Photoinduced Solid-to-Liquid Transitions of Azopolymers

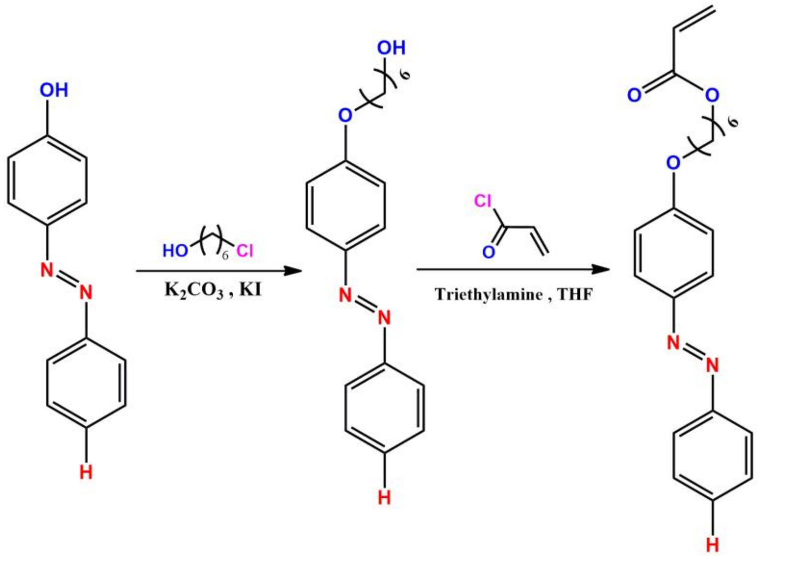

Figure S1. Scheme for synthesis of monomer M-1-Azo.

Synthesis of monomer M-1-Azo: 4-hydroxyazobenzene (1.98 g, 10 mmol) was dissolved in 20 mL of DMF, and 3.45 g of K_2CO_3, 0.05 g of KI was added and stirred well; further, 6-bromo-1-hexanol (2.17 g, 12 mmol) was added dropwisely, and the reaction was carried out at 110 °C for 12 h. After cooling, 200 mL of deionized water was added, and a yellow precipitate was precipitated. Extraction, column separation (petroleum ether: ethyl acetate = 3:1) gave compound 2.

Compound 2 (2.98 g, 10 mmol) was dissolved in 100 mL of THF, and triethylamine (3 g, 30 mmol) was added and stirred. Acryloyl chloride (2.7 g, 30 mmol) was added and the reaction was carried out at 0 °C for 2 h. Filter and then remove the solvent, column chromatography (petroleum ether: ethyl acetate = 10:1) gave an orange solid of compound 3 (M-1-Azo).

1H NMR (400 MHz, Chloroform-d) δ 7.92 (dd, J = 18.3, 8.1 Hz, 4H), 7.50 (t, J = 7.4 Hz, 2H), 7.45 (d, J = 7.1 Hz, 1H), 7.01 (d, J = 8.8 Hz, 2H), 6.41 (d, J = 17.3 Hz, 1H), 6.13 (dd, J = 17.3, 10.4 Hz, 1H), 5.83 (d, J = 10.4 Hz, 1H), 4.19 (t, J = 6.6 Hz, 2H), 4.06 (t, J = 6.4 Hz, 2H), 1.89 – 1.81 (m, 2H), 1.74 (d, J = 7.6 Hz, 2H), 1.56 – 1.45 (m, 4H).

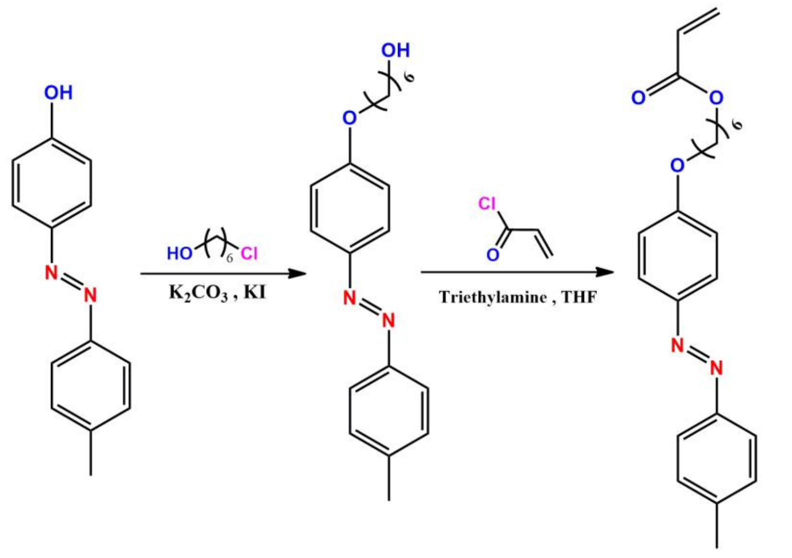

Figure S2. Scheme for synthesis of monomer M-2-Azo.

Synthesis of monomer M-2-Azo: 4-Methyl-4′-hydroxyazobenzene (2.12 g, 10 mmol) was dissolved in 20 mL of DMF, and 3.45 g of K_2CO_3, 0.05 g of KI was added and stirred well; further, 6-bromo-1-hexanol (2.17 g, 12 mmol) was added slowly, and the reaction was carried out at 110 °C for 12 h. After cooling, 200 mL of deionized water was added, and a yellow precipitate was precipitated. Extraction, column separation (petroleum ether: ethyl acetate = 3:1) gave compound 5.

Compound 5(3.13 g, 10mmol) was dissolved in 100 mL of THF, and triethylamine (3 g, 30 mmol) was added and stirred. Acryloyl chloride (2.7 g, 30 mmol) was added and the reaction was carried out at 0 ° C for 2 h. Filter and then remove the solvent, column chromatography (petroleum ether: ethyl acetate = 10:1) gave an orange solid of compound 6 (M-2-Azo).

^{1}H NMR (400 MHz, Chloroform-*d*) δ 7.93 (d, *J* = 8.6 Hz, 2H), 7.81 (d, *J* = 8.0 Hz, 2H), 7.30 (d, *J* = 8.0 Hz, 2H), 7.00 (d, *J* = 8.8 Hz, 2H), 6.41 (d, *J* = 17.3 Hz, 1H), 6.13 (dd, *J* = 17.3, 10.4 Hz, 1H), 5.82 (d, *J* = 10.4 Hz, 1H), 4.18 (t, *J* = 6.6 Hz, 2H), 4.05 (t, *J* = 6.4 Hz, 2H), 2.43 (s, 3H), 1.90 – 1.79 (m, 2H), 1.76 – 1.68 (m, 2H), 1.57 – 1.44 (m, 4H).

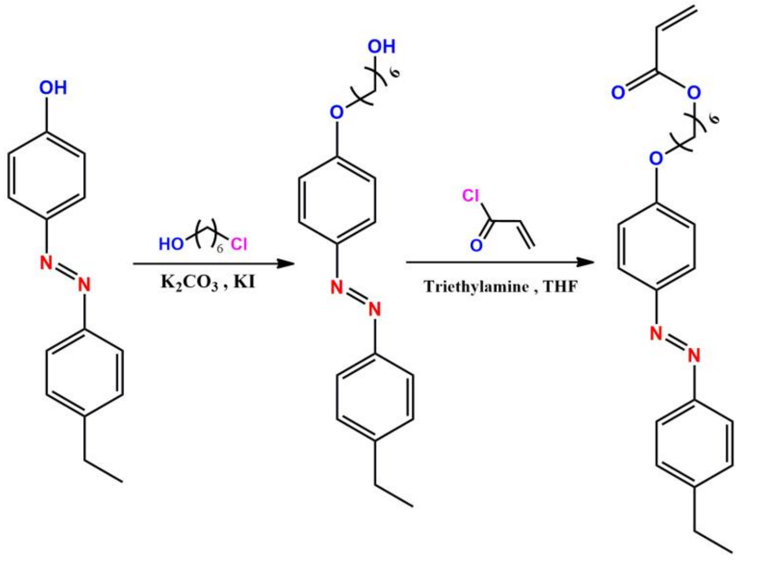

Figure S3. Scheme for synthesis of monomer M-3-Azo.

Synthesis of monomer M-3-Azo: 4-Ethyl-4′-hydroxyazobenzene (2.26 g, 10 mmol) was dissolved in 20 mL of DMF, and 3.45 g of K_2CO_3, 0.05 g of KI was added and stirred well; further, 6-bromo-1-hexanol (2.17 g, 12 mmol) was added slowly, and the reaction was carried out at 110 °C for 12 h. After cooling, 200 mL of deionized water was added, and a yellow precipitate was precipitated. Extraction, column separation (petroleum ether: ethyl acetate = 3:1) gave compound 8.

Compound 8 (3.26 g, 10 mmol) was dissolved in 100 mL of THF, and triethylamine (3 g, 30 mmol) was added and stirred. Acryloyl chloride (2.7 g, 30 mmol) was added and the reaction was carried out at 0 ° C for 2 h. Filter and then remove the solvent, column chromatography (petroleum ether: ethyl acetate = 10:1) gave an orange solid of compound 9 (M-3-Azo).

^{1}H NMR (400 MHz, Chloroform-d) δ 7.93 (d, J = 8.9 Hz, 2H), 7.83 (d, J = 8.3 Hz, 2H), 7.32 (d, J = 8.3 Hz, 2H), 7.00 (d, J = 8.9 Hz, 2H), 6.41 (d, J = 17.3 Hz, 1H), 6.13 (dd, J = 17.3, 10.4 Hz, 1H), 5.82 (d, J = 11.7 Hz, 1H), 4.18 (t, J = 6.6 Hz, 2H), 4.05 (t, J = 6.4 Hz, 2H), 2.72 (q, J = 7.6 Hz, 2H), 1.89 – 1.79 (m, 2H), 1.77 – 1.68 (m, 2H), 1.58 – 1.45 (m, 4H), 1.29 (d, J = 7.6 Hz, 3H).

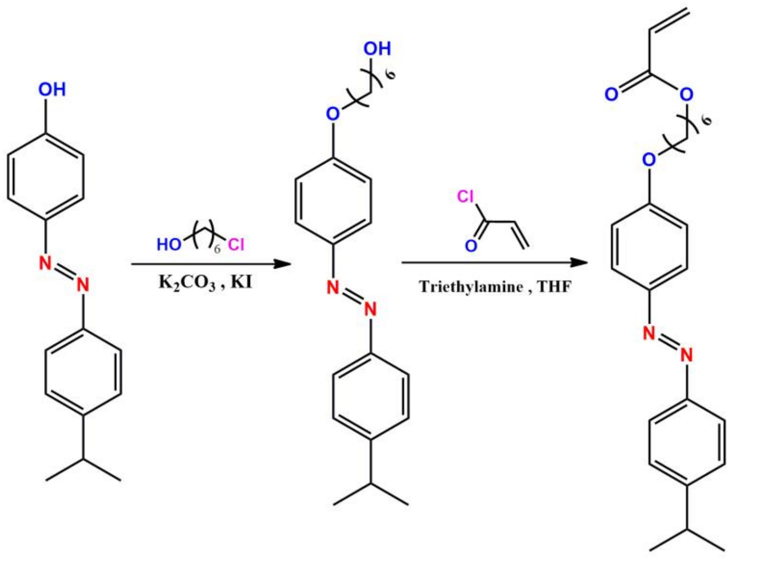

10 11 12 (M-4-Azo)

Figure S4. Scheme for synthesis of monomer M-4-Azo.

Synthesis of monomer M-4-Azo: 4-Isopropyl-4′-hydroxyazobenzene (2.40 g, 10 mmol) was dissolved in 20 mL of DMF, and 3.45 g of K_2CO_3, 0.05 g of KI was added and stirred well; further, 6-bromo-1-hexanol (2.17 g, 12 mmol) was added slowly, and the reaction was carried out at 110 °C for 12 h. After cooling, 200 mL of deionized water was added, and a yellow precipitate was precipitated. Extraction, column separation (petroleum ether: ethyl acetate = 3:1) gave compound 11.

Compound 11 (3.40 g, 10 mmol) was dissolved in 100 mL of THF, and triethylamine (3 g, 30 mmol) was added and stirred. Acryloyl chloride (2.7 g, 30 mmol) was added and the reaction was carried out at 0 ° C for 2 h. Filter and then remove the solvent, column chromatography (petroleum ether: ethyl acetate = 10:1) gave an orange solid of compound 12 (M-4-Azo).

^{1}H NMR (400 MHz, Chloroform-d) δ 8.01 (d, J = 8.8 Hz, 2H), 7.90 (d, J = 8.4 Hz, 2H), 7.36 (d, J = 8.4 Hz, 2H), 7.01 (d, J = 9.0 Hz, 2H), 6.41 (d, J = 17.3 Hz, 1H), 6.13 (dd, J = 17.3, 10.4 Hz, 1H), 5.83 (d, J = 10.4 Hz, 1H), 4.18 (t, J = 6.6 Hz, 2H), 4.06 (t, J = 6.4 Hz, 2H), 2.99 (p, J = 6.7 Hz, 1H), 1.88 – 1.81 (m, 2H), 1.77 – 1.69 (m, 2H), 1.57 – 1.45 (m, 4H), 1.30 (d, J = 6.9 Hz, 6H).

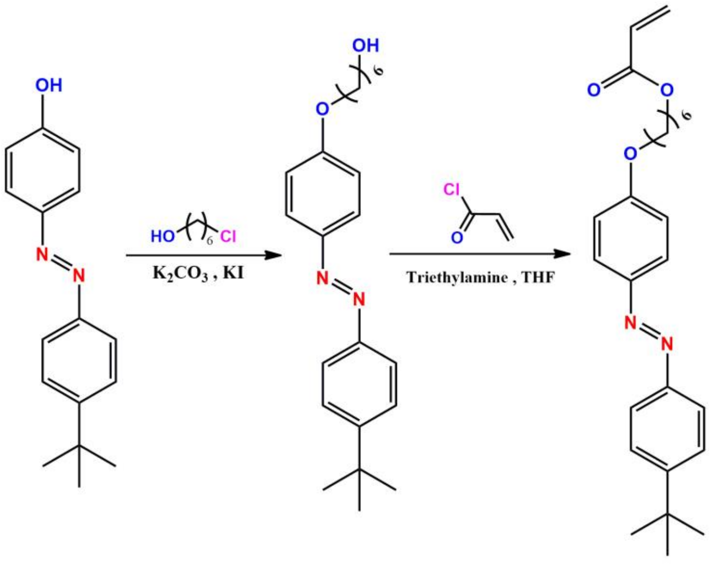

Figure S5. Scheme for synthesis of monomer M-5-Azo.

Synthesis of monomer M-5-Azo: 4-tert-butyl-4′-hydroxy azobenzene (2.54 g, 10 mmol) was dissolved in 20 mL of DMF, and 3.45 g of K_2CO_3, 0.05 g of KI was added and stirred well; further, 6-bromo-1-hexanol (2.17 g, 12 mmol) was added slowly, and the reaction was carried out at 110 °C for 12 h. After cooling, 200 mL of deionized water was added, and a yellow precipitate was precipitated. Extraction, column separation (petroleum ether: ethyl acetate = 3:1) gave compound 14.

Compound 14 (3.54 g, 10 mmol) was dissolved in 100 mL of THF, and triethylamine (3 g, 30 mmol) was added and stirred. Acryloyl chloride (2.7 g, 30 mmol) was added and the reaction was carried out at 0 °C for 2 h. Filter and then remove the solvent，column chromatography (petroleum ether: ethyl acetate = 10:1) gave an orange solid of compound 15 (M-5-Azo).

^1H NMR (400 MHz, Chloroform-d) δ 7.95 (d, J = 8.8 Hz, 2H), 7.85 (d, J = 8.5 Hz, 2H), 7.52 (d, J = 8.6 Hz, 2H), 7.00 (d, J = 8.9 Hz, 2H), 6.41 (d, J = 17.3 Hz, 1H), 6.13 (dd, J = 17.3, 10.4 Hz, 1H), 5.82 (d, J = 11.6 Hz, 1H), 4.18 (t, J = 6.6 Hz, 2H), 4.05 (t, J = 6.4 Hz, 2H), 1.89 – 1.80 (m, 2H), 1.77 – 1.69 (m, 2H), 1.57 – 1.44 (m, 4H), 1.37 (s, 9H).
